# TRIM family contribute to tumorigenesis, cancer development, and drug resistance

**DOI:** 10.1186/s40164-022-00322-w

**Published:** 2022-10-19

**Authors:** Ning Huang, Xiaolin Sun, Peng Li, Xin liu, Xuemei Zhang, Qian Chen, Hong Xin

**Affiliations:** 1 Department of Pharmacology, School of Pharmacy & General Surgery of Minhang Hospital, Fudan University, Shanghai, 201203 China; 2 PharmaLegacy Laboratories Co.,Ltd, Shengrong Road No.388, Zhangjiang High-tech Park, Pudong New Area, Shanghai, China

**Keywords:** TRIM family, Cancer, Tumorigenesis, Cancer resistance, Signaling pathway, EMT, CSCs

## Abstract

The tripartite-motif (TRIM) family represents one of the largest classes of putative single protein RING-finger E3 ubiquitin ligases. TRIM family is involved in a variety of cellular signaling transductions and biological processes. TRIM family also contributes to cancer initiation, progress, and therapy resistance, exhibiting oncogenic and tumor-suppressive functions in different human cancer types. Moreover, TRIM family members have great potential to serve as biomarkers for cancer diagnosis and prognosis. In this review, we focus on the specific mechanisms of the participation of TRIM family members in tumorigenesis, and cancer development including interacting with dysregulated signaling pathways such as JAK/STAT, PI3K/AKT, TGF-β, NF-κB, Wnt/β-catenin, and p53 hub. In addition, many studies have demonstrated that the TRIM family are related to tumor resistance; modulate the epithelial–mesenchymal transition (EMT) process, and guarantee the acquisition of cancer stem cells (CSCs) phenotype. In the end, we havediscussed the potential of TRIM family members for cancer therapeutic targets.

## Introduction

Considerable progress has been made in elucidating cancer pathological mechanisms, and oncological therapeutic research, yet, multiple obstructions, such as tumor resistance still remain. Uncovering the underlying causes of tumorigenesis and cancer development may help resolve these obstacles and discover potential drug targets. The tripartite motif (TRIM)-containing protein family proteins were characterized as E3 ubiquitin ligases and were first known for their involvement in innate immunity as pathogen-recognition effectors and regulators in transcriptional pathways [1]. In 2011, Shigetsugu Hatakeyama preliminarily discussed the roles of some TRIM family members in specific tumorigenic pathways and cellular stress response regulation, indicating their diverse contributions to tumor development [2]. Over the last decade, many studies on the roles of TRIM family members in cancer have been published, providing us with a more comprehensive and detailed understanding of these proteins.

## TRIM family structure and functions

The TRIM family is composed of an N-terminal RING domain, one or two B-box motifs, and the alpha-helical coiled-coil domain followed by a highly variable carboxyl-terminal domain from the N-terminal to the C-terminal. The RING domain constitutes the catalytic center, which is a unique linear series of cysteine and histidine residues of a zinc finger domain. The RING domain exerts its role by mediating protein–protein interactions. The dimerization of RING is often a prerequisite for ubiquitin ligase activity. Usually, the TRIM family differs in the C-terminus. B-box motifs are composed of small peptide sequences containing finger-like protrusions that participate in target protein recognition. The coiled-coil region is usually involved in TRIM homo- or oligomerization [3].

**Fig. 1:**
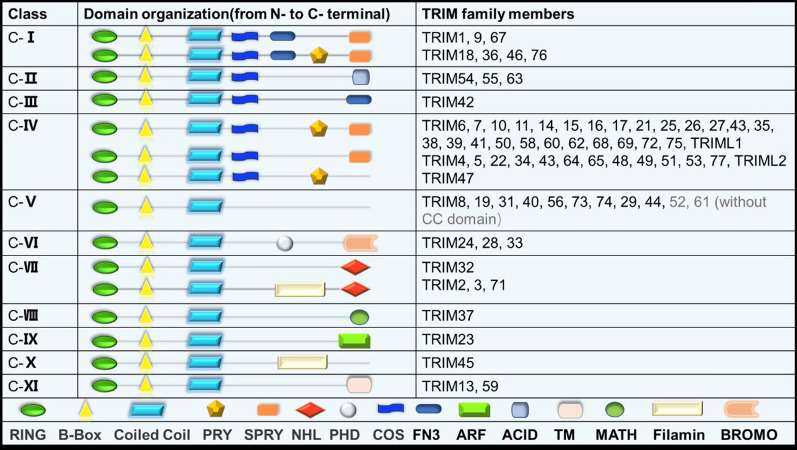
Structural classification of TRIM family members. The majority of TRIM proteins contain an N-terminal RING domain, one or two B-box domains (B1, B2) and a coiled-coil domain (CC) and are classified into 11 subfamilies (C-I-C-XI) based on a variable C-terminal domain. There is an additional unclassified group lacking a RING domain. *SPRY* named from SPla and the RYanodine receptor, *PRY-SPRY* associated domain, NHL-NCL1, HT2A and LIN41 domain, *PHD* plant homeodomain, *COS* cos-box, *FN3* fibronectin type III, ARF-*ADP* ribosylation factor domain, *ACID* acid-rich region, *TM* transmembrane region, *MATH* meprin and TRAF-homology domain, Filamin-filamin-type I G domain, BROMO-bromodomain

**Fig.2:**
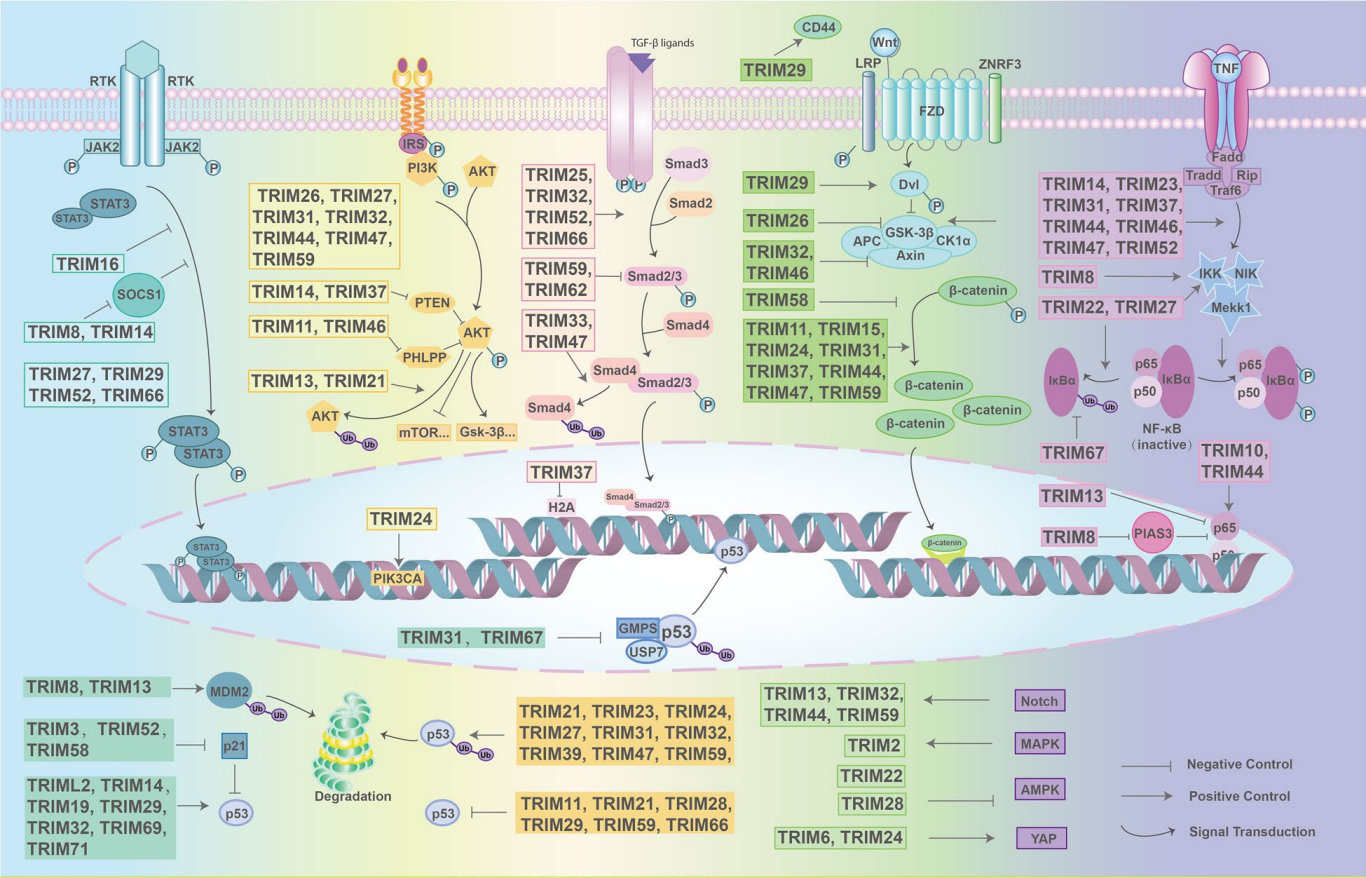
TRIM family members are involved in cancer development. TRIM family members are involved in cancer development through mediating the abnormal cellular signaling pathway transduction including JAK/STAT, PI3K/AKT, TGF-β, Wnt/β-catenin, NF-κB, p53 hub, and other signaling pathways such as Notch, MAPK, AMPK, YAP. TRIMs affect upstream factors and downstream effectors through positive regulations and negative regulations which are mainly achieved by exerting their E3 ligase activity and promoting the ubiquitination degradation of target proteins. By interfering these crucial signaling pathways, TRIMs influence proliferation, migration, and invasion characteristics of cancer cells

TRIM proteins are ubiquitously expressed. After a long evolutionary history, the tripartite structure is highly conserved and exclusive to metazoans and varies based on the type of TRIM proteins [4]. TRIMs comprise a large protein family: to date in excess of 80 proteins. The subdivision of TRIMs is diverse. According to the highly variable carboxy-terminal domain, TRIMs with RING domain can be categorized into 11 distinct subgroups (C-I-C-XI, listed in Fig. 1) [3]. There are eight TRIM members with PYRIN domain instead of the RING domain remain unclassified. The variable domains in the C-terminal portion of TRIM proteins constitute the functional units that mediate target recognition and specificity Fig. 2. The most common C-terminal domain is PRY-SPRY. Approximately half of the TRIM family subgroups C-IV are characterized by a PRY-SPRY domain. SPRY is first identified in the SP1A kinase of Dictyostelium and rabbit Ryanodine receptor, PRY refers to SPRY-associated domain. PRY-SPRY mainly mediates protein–protein interactions and is present in TRIM proteins either alone or preceded by COS (cos-box), and FN3 (fibronectin type III) domains [5]. Although the combination and the order of domains may differ, the spacing between each domain is highly conserved. Various combinations result in different specialized functions [6].

Many reviews have noted that TRIM family members are engaged in a highly diverse cellular activities and processes, including RNA-binding activities such as mRNA repression and mRNA localization [7], autophagy [8, 9], pyroptosis, apoptosis [10], cell cycle progression and mitosis [11], DNA damage responses [12], viral infections [13], immune activation and inflammatory processes [14, 15]. In addition, TRIMs also control critical signaling pathways and hub protein activity by acting as ubiquitination effectors and ubiquitin-like modifiers.

**Fig.3:**
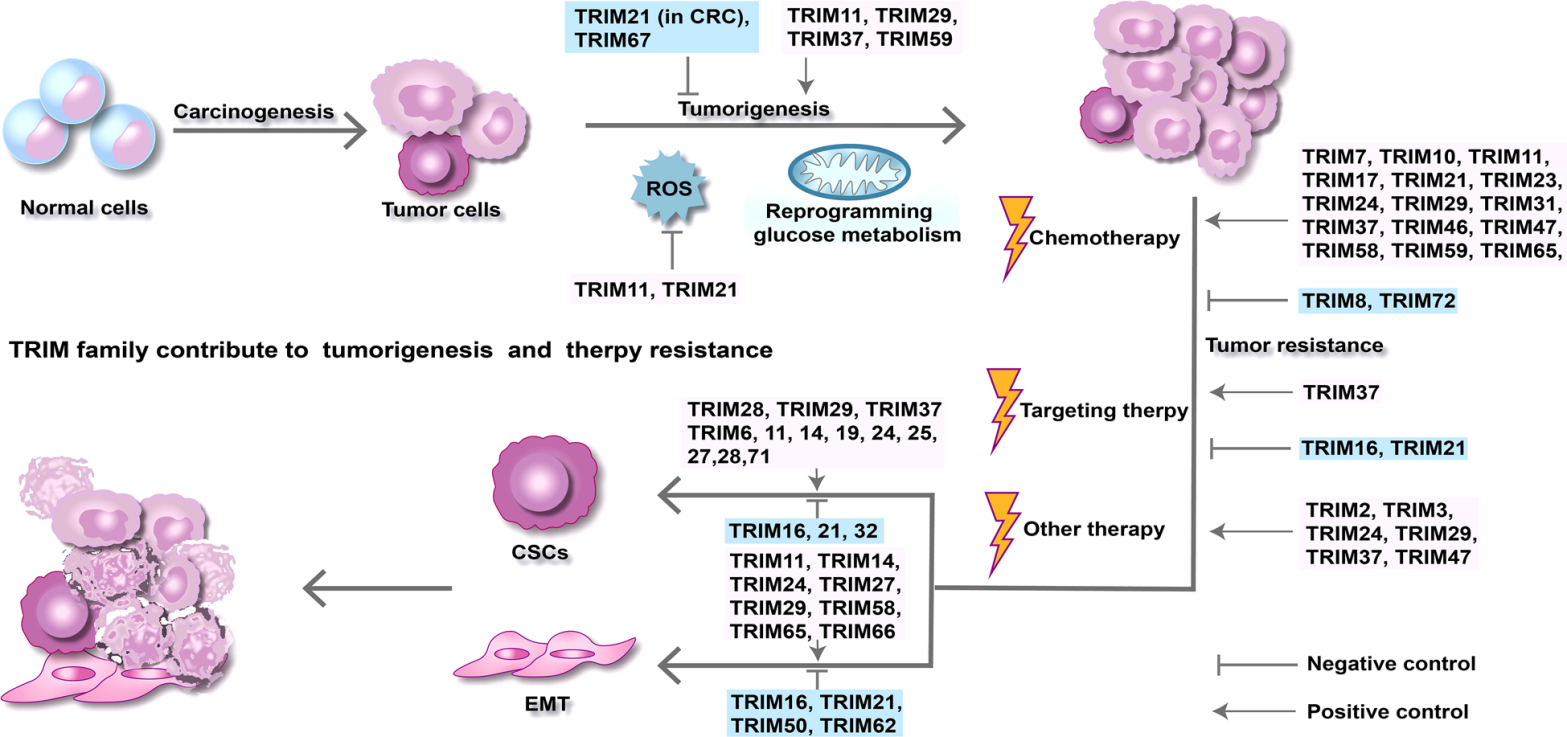
TRIM family are involved in tumor resistance. At the pre-stage of tumorigenesis, a tiny subset of normal cells undergoes genomic changes to transform into malignant tumor cells. Subsequently, beneficial factors including metabolic reprogramming and ROS contribute to the clonal expansion of tumor cells. Although anti-tumor treatments have been administrated, some tumor cells escape death through all kinds of mechanisms including CSCs phenotypes acquisition and the EMT process. TRIM family functions as different roles in tumorigenesis, modulating the EMT process and CSCs maintenance and affecting cancer therapy sensitivity

Ubiquitination is a fundamental and versatile post-translational modification that plays a crucial role in cellular processes often resulting in targeted degradation of conjugated proteins. Fig .3 Ubiquitin is a 76-residue polypeptide. Attachment of polyubiquitin chains requires the activities of an E1 activating enzyme, an E2 ubiquitin-conjugating enzyme, and an E3 ubiquitin protein ligase that specifically recognizes the substrate. It binds with the E2 enzyme and facilitates the covalent binding of ubiquitin [16]. E3 ubiquitin proteins consists of three protein families: RING E3s, HECT E3s, and RBR E3s. As we mentioned above, most members of the TRIM family have a RING domain, which can bind to a ubiquitin-loaded E2 enzyme and promote the transfer of ubiquitin to a target protein. Hence, most TRIM members have been identified to function as E3 ubiquitin ligases. The fates of the substrate proteins depend upon the lysine of the ubiquitin moiety used for isopeptide bond formation. The most predominant linkage type is Lys48-linked chains, which target proteins to the proteasome for degradation [17]. In contrast, the second most abundant type linked via Lys63 performs has nondegradative roles [18]. In addition, the length of the ubiquitin chain, and the type of ubiquitin-like molecule are important. Thus, TRIM proteins may have a “nonproteolytic” role of ubiquitination. In short, the function of TRIMs and the fate of the target proteins depend on many factors.

Another common post-translational modification, SUMOylation, is a process similar to ubiquitination. The difference is that small ubiquitin-like modifier (SUMO) proteins are covalently conjugated to the target proteins; The subsequent fate also differs: SUMOylation usually alters the properties of target proteins, such as stability, activity, cellular localization, and protein–protein interactions, rather than degradation [19]. However, SUMO enzymes are fewer in number than their counterparts. Of note, the present research suggests that TRIM proteins constitute a new category of SUMO E3. For example, TRIM19, and TRIM27 exhibit SUMO E3 ligase activity in the MDM2-p53 axis [20]. The SUMO enzyme activity of TRIMs may also better explain their diverse functions. Their ubiquitination or ubiquitin-like activity direct the target proteins to different destiny: degradation at the proteasome level, stabilization or relocalization in corresponding cellular compartments [21].

## The TRIM family and cancer pathology

Given the role of the TRIM family as crucial post-translational protein modifiers, effectors of the cell signaling pathway, and regulatory genes, it is not surprising that distinct TRIM family members exert diverse influences on cancer cell phenotypes, modulating proliferation, migration, and invasion. Various TRIM family genes have been identified to be significantly under- or overexpressed in cancer metastases compared to primary tumors in different cancers [22, 23]. In some cases, the expression levels of TRIMs could be biomarkers and prognostic factors of cancers. A study profiled the expression patterns of the TRIM family in cancer and an interesting phenomenon was found: the expression of 10 TRIM genes, TRIM3, TRIM7, TRIM14, TRIM16, TRIM21, TRIM22, TRIM29, TRIM59, TRIM66, and TRIM70, was significantly upregulated in NSCLC cell lines compared with the normal cell line, whereas the expression of 7 other TRIM genes, TRIM4, TRIM9, TRIM36, TRIM46, TRIM54, TRIM67, and TRIM76, was significantly downregulated [24]. To date, the involvement of TRIM proteins in cancer prognosis have been summarized in many articles according to the diverse tumor types. Table 1 summarizes the relationship between the expression levels of TRIM family members and adverse prognosis in various types of clinical tumor tissue samples.

**Table 1:** The expression levels of TRIM family proteins related to the adverse prognostic

Cancer type	Low expression level	High expression level
AML	TRIM62 [23]	/
HCC	TRIM26 [24], TRIM28, TRIM37, TRIM45, TRIM59 [25], TRIM59 [26]	TRIM11 [27, 28], TRIM16 [29], TRIM24 [30], TRIM25 [31], TRIM27 [32], TRIM31 [33], TRIM37 [34], TRIM50 [26], TRIM3, TRIM5, TRIM21, TRIM27, TRIM32, TRIM44, TRIM47, TRIM72 [35]
RCC	/	TRIM27 [36], TRIM37 [37], TRIM44 [38]
UCB	/	TRIM65 [39]
GBC		TRIM31 [40]
PTC	/	TRIM14 [41]
HEC	/	TRIM44 [42]
TGCT	/	TRIM44 [43]
NPC	/	TRIM11 [44]
OC	TRIM50 [45]	TRIM59 [46]
CRC	TRIM1, TRIM2, TRIM6, TRIM13, TRIM26, TRIM35,TRIM55 [47], TRIM58 [48]	TRIM23 [49], TRIM24 [50], TRIM27 [51], TRIM47 [47], TRIM39 [52]
Pancreatic carcinoma	TRIM36 [53], TRIM50 [54]	TRIM11 [55], TRIM15 [56], TRIM37 [57], TRIM47 [58], TRIM59 [59]
Gastric carcinoma	TRIM3 [60], TRIM21 [61]	TRIM14 [62], TRIM15 [63], TRIM23 [64], TRIM29 [65], TRIM32 [66, 67], TRIM44 [68], TRIM47 [69], TRIM54 [70]
Glioblastoma	TRIM8 [71], TRIM16 [72], TRIM17 [73]	TRIM14 [74], TRIM21 [75], TRIM28 [76]
Breast carcinoma	TRIM16 [72], TRIM29 [77], TRIM31 [78], TRIM35 [79]	TRIM3 [80], TRIM39 [81], TRIM44 [82, 83], TRIM47 [84], TRIM59 [85, 86], TRIM62 [87]
Lung carcinoma	TRIM28 [88], TRIMs56 [89]	TRIM11 [90], TRIM15 [91, 92], TRIM23 [93], TRIM35 [94], TRIM46 [95], TRIM66 [96]
melanoma	/	TRIM27 [97]
Squamous carcinoma	/	TRIM28 [98]
Osteosarcoma	TRIM22 [99]	TRIM7 [100]

The expression levels of different TRIM family proteins positively related to the adverse prognostic in various types of clinical tumor tissue samples. The adverse prognostic refers to the lower survivability of the diagnosis

*AML*: acute myelocytic leukemia, *HCC*: hepatocellular carcinoma, *RCC* renal cell carcinoma, *UCB*: urothelium carcinoma, *PTC*: papillary thyroid cancer, *LC* lung cancer, *HEC*: human esophageal cancer, *LUAD* lung adenocarcinoma, *TGCT* testicular germ cell tumors, *NPC* nasopharyngeal carcinoma; *OC* ovarian cancer, *CRC* colorectal cancer

Recently, the potential of TRIM family members as biomarkers in early detection and diagnosis has been evaluated. Increased TRIM59 expression and protein levels were observed in CRC tissues and benign colonic lesions compared to nontumor tissues. Moreover, the expression levels of TRIM59 had significant interrelations with tumor, node, and metastasis staging [103]. TRIM23 was also upregulated and associated with tumor size, lymph node metastasis, stage, and poor prognosis in CRC [51]. TRIM29 also served as an independent predictor for lymph nodes in gastric cancer [67]. TRIM62, was identified as a new gene mapping to a region of high-frequency loss of heterozygosity (LOH) in diverse cancers. In addition, during the transition to ductal carcinoma in situ (DCIS), which represents an early histological stage in breast tumorigenesis, TRIM62 expression was downregulated [89]. Early detection of cancer would benefit patients because early detection makes tumors more resectable and treatment more efficacious. The potential of TRIM family members as biomarkers in the early detection, diagnosis, and treatment of benign colonic lesions has been gradually recognized. While the complexity varies by cancer type, more effective biomarkers and comprehensive integration of various biomarkers are needed. Therefore, a specific understanding of the involvement of TRIMs in early tumor initiation may provide new biomarkers for efficient diagnosis.

### The TRIM family participates in tumorigenesis

Tumorigenesis still represents one of the most intriguing and intricate research subjects. At the pre-stage of tumorigenesis, a small subset of randomly occurring genomic changes confers advantageous cell phenotypes. Subsequently, a series of beneficial factors including metabolic reprogramming, tumor microenvironment (TME) changes, reactive oxygen species (ROS) dysregulation, and aberrant inflammasome signaling, promote malignant transformation and finally lead to the clonal expansion of the cells that express these phenotypes [104].

#### ROS dysregulation

Dysregulation of ROS is a common feature of tumor cells. ROS play a vital role in modulating the tumor microenvironment, affecting the different stromal cells that provide metabolic support, blood supply, and immune responses. ROS influence tumor initiation and transformation in apparently contradictory ways, and the underlying mechanisms are complicated. Therefore, the effects of ROS-modulating therapies are difficult to predict. A better understanding of the complexity of ROS in cancer will help elucidate the potential of ROS-targeting therapies for cancer treatment [105, 106].

TRIM family members have been reported to sustain cellular homeostasis balance under oxidative stress. For example, recombinant human MG53, namely TRIM72, protected hair follicle stem cells from oxidative stress-induced apoptosis [107]. ROS can upregulate TRIM37 during HBV (Hepatitis B)-associated hepatic fibrosis which is a high risk factor for hepatocellular carcinoma [108]. Under a hypoxic conditions, TRIM14 was induced by sponging miR-191-5p in colon cancer cells, inhibiting apoptosis and promoting proliferation and invasion [109]. Some TRIMs may play opposite roles: TRIM4 could sensitize cells to hydrogen peroxide (H_2_O_2_) induced cell death [110]. Similarly, knockdown of TRIM8 can alleviate hypoxia/reoxygenation-induced oxidative stress [111]. Nuclear factor erythroid 2-related factor 2 (Nrf2), a crucial cellular redox regulator, has been identified as a novel target for TRIM family proteins. In osteosarcoma, TRIM22 can interact with Nrf2 and accelerate its degradation by inducing ubiquitination dependent on its E3 ligase activity, thus activating downstream AMPK/mTOR signaling, and affecting Nrf2-mediated ROS imbalance. Moreover, TRIM22 was found to inhibit the Warburg effect in osteosarcoma cells [101]. In another study, TRIM15 was identified to promote NSCLC progression via Nrf2 stability mediated by promoting Keap1 (Kelch-like ECH associated protein 1) ubiquitination and degradation [94]. Keap1 is an E3 ubiquitin ligase, which tightly regulates the activity of Nrf2 by targeting it for ubiquitination. TRIM16 is also an integral part of the p62-Keap1-Nrf2 complex and utilizes multiple mechanisms to stabilizing Nrf2 [112]. Tumor cells also exhibit a powerful capacity, such as augmented antioxidant defense, to maintain endoplasmic reticulum (ER) homeostasis. A study found that TRIM25 is significantly induced and relieves oxidative stress upon ER stress, promoting tumor cell growth in vitro and in vivo. Mechanistically, TRIM25 directly targets Keap1 by ubiquitination and degradation which leads to Nrf2 activation [33].

To date, only a few articles have further discussed the relationship between the regulation of cellular oxidative pressure by TRIM family proteins and tumor initiation. One study found that TRIM11 promotes tumorigenesis and is regulated by Nrf2—one of the mechanisms is reducing oxidative stress during oncogenic growth [113]. The involvement of TRIM21 in the p62-Keap1-Nrf2 axis has been reported before [114–116]. Recently, TRIM21- knockout mice were identified to be resistant to DEN-induced hepatocarcinogenesis because the genetic ablation of TRIM21 protected cancer cells from oxidative hepatic damage [117]. Further research found that RPRD1A (regulation of nuclear pre-MRNA domain containing 1A) competitively interacts with TRIM21, affecting the upregulation of the P62-Keap1-Nrf2 pathway, and enhancing gene expression to counteract oxidative stress, maintain cancer cell survival, and promote HCC development [118].

Whereas the damaging effects of ROS can be detrimental to cell survival, the enhanced ROS driven by oncogenic perturbations is a characteristic of cancer cells as ROS drive the accumulation of oncogenic alterations that promote cancer development. Moreover, the capacity to clear oxidative substances ensures cancer cell cellular homeostasis, promoting their development. Inhibition of the way that ROS contribute to tumorigenicity and tumor development by interfering with TRIM family proteins may be a strategy, although detailed studies are needed.

#### Reprogramming glucose metabolism

A growing amount of evidence has shown that initial cancer cells undergo metabolic reprogramming including glucose metabolism, which is mediated by oncogenic drivers and by the undifferentiated character of tumor cells. The “Warburg effect”, defined as the observation that cancer cells increase the rate of glucose uptake and prefer glycolysis rather than oxidative phosphorylation even in the presence of oxygen, is widely accepted as one of the reprogramming glucose metabolism [119–121]. Besides, high rates of glucose metabolism is also maintained in cancer cells [122]. Tumorigenesis is evidently dependent on tumor-associated metabolic reprogramming: the excessive intake of nutrients and their redistribution to metabolic pathways that are conducive to cellular tumorigenic properties, determine cellular fate and affect the tumor microenvironment, which is involved in selecting malignant phenotypes.

Several reports have shown that TRIMs participate in cancer cell reprogramming through different mechanisms. Transformed NSCLC cells maintained high glycolytic rates regardless of changing environmental mechanics by downregulating TRIM21 and by sequestering residual TRIM21 on a stress-fiber subset to retain phosphofructokinase expression and to achieve mechanical regulation of glycolysis [123]. The knockdown of TRIM23 in LUAD cells inhibited cell proliferation in vitro and in vivo, GLUT1/3 (glucose transporter type 1/3) expression, glucose uptake, and lactate and ATP production. Further research found that TRIM23 acted as an oncogene by regulating glucose metabolism via the NF-κB/GLUT1/3 axis [95]. Similarly, TRIM32 promoted the growth of gastric cancer cells through by enhancing glucose transportation by targeting GLUT1 [69]. The ubiquitous AKT has diverse downstream effects on cellular metabolism, via either direct regulation of nutrient transporters and metabolic enzymes or the control of transcription factors that regulate the key components. TRIM27 was reported to accelerate the glucose uptake of ESCC cells by mediating the polyubiquitination of PTEN and activating PTEN-AKT pathway [124]. To conclude, TRIM proteins are involved in tumor cell glucose metabolism reprogramming and may participate in the early stage of tumor initiation. These discoveries may shed a light on the specific component function in tumor cell “developmental pliancy” and contribute to development of other novel therapeutics.

#### Other mechanisms

To date, there are multiple reports that refer to the involvement of TRIM family proteins in tumorigenesis. Several studies have investigated the modulatory effects of TRIMs on oncogenic proteins or drug-induced cancer: the TRIM59 gene was upregulated in SV40 (simian vacuolating virus 40) Tag oncogene-directed transgenic and knockout mouse prostate cancer models. Further research found that the p-Ser/Thr modification of TRIM59 is correlated with tumorigenesis and that p-Tyr-TRIM59 is correlated with advanced cancer of the prostate. ShRNA-mediated knockdown of the TRIM59 caused S-phase cell cycle arrest and cell growth retardation. Moreover, the upregulation of TRIM59 gene coincided with the upregulation of genes specific to the Ras signaling pathway and bridging genes for SV40 Tag-mediated oncogenesis [125]. A study revealed that, in pancreatic ductal adenocarcinoma (PDA) models, TRIM29 accelerated pancreatic intraepithelial neoplasia (PanIN) formation and promoted the development of invasive and metastatic PDA cancer in the presence of oncogenic KRAS [126]. In contrast, TRIM67 functions as a pivotal tumor suppressor in colorectal cancer: colon-specific knockout of TRIM67 significantly accelerates azoxymethane-induced colorectal cancer in mice by activating the p53 signaling pathway [127]. A recent study found that TRIM21 was decreased in CRC and ulcerative colitis (UC)-associated cancer and negatively regulated intestinal epithelial carcinogenesis by modulating epithelial cancer cell proliferation, adhesion, tissue remodeling, angiogenesis, and proinflammatory responses [128]. Ectopic TRIM37 expression induced tumorigenicity in nontransformed cells. Further study revealed the underlying mechanism: TRIM37 could mono-ubiquitinate histone H2A, leading to the silencing of tumor suppressor genes, and facilitating the transformation from normal cells to tumor cells [129]. A recent study also confirmed this conclusion in RCC tumors. Moreover, the upregulated expression of TRIM37 could predict aggressive neoplastic phenotypes [39]. In addition, knockdown of TRIM11 suppressed the tumorigenicity of chordoma cells [130]. Because of the capability of some TRIMs to ubiquitinate misfolded proteins, we wondered whether this effect could play a role in tumorigenesis. Misfolded proteins are continually produced in cells, and are degraded mainly through autophagy and ubiquitin–proteasome systems. The diminished capability to degrade misfolded proteins is associated with a multitude of diseases, such as neurodegenerative diseases. However, the outcome of an enhanced capacity remains unclear. Interestingly, research showed that this augmented capacity was required and associated with the initiation and maintenance of malignant phenotypes. The higher degradation power in cancer cells could be attributed to enhanced proteasome activity and especially TRIM proteins. A study investigated whether TRIM11 promoted tumorigenesis through its ability to accelerate the degradation of misfolded proteins [113]. TRIM11, TRIM19, TRIM21, TRIM5, TRIM16, TRIM13, and TRIM25 also exhibited a strong capacity for misfolded protein removal. However, the specific investigations of their capability and proteasome-related oncogenesis are rare [131].

In brief, the clear recognition of TRIMs in tumor initiation, as well as the importance of TRIMs as tumor biomarkers and prognostic factors, is required to better understand cancer pathology and provide precision diagnostics and prognostic analysis for patients. Thus, investigations of metabolism not only benefit the understanding of carcinogenesis and cancer progression but also provide new insights into cancer treatments.

### TRIM family members are involved in cancer development

The contribution of TRIM family members to various cancers has been widely reported. Identification of the role of the TRIM family members should be discussed in the context of specific conditions. Whether TRIM family proteins act as oncogenes or tumor suppressors depends on the tumor type and specific signaling pathway. Here, we characterized the regulatory networks of TRIMs involved in various signaling pathways in different cancers and summarized in Fig. 2.

#### p53 hub

As p53 is one of the most crucial tumor-suppressor proteins, abnormalities such as mutations and abnormal expression levels of p53 are observed in the majority of human tumors [132]. p53 is involved in the transcription of genes, cell cycle control, DNA repair, induction of apoptosis, cell senescence and programmed death, and regulation of cellular metabolism [133, 134]. The expression of the tumor suppressor p53 is regulated at multiple levels, the disruption of which often leads to cancer. The stabilization of p53 is usually achieved by increasing the protein half-life and activating the transcriptional function of p53. MDM2 (mouse doubleminute 2 homolog) is a vital regulator of p53 by three different interconnected modes. MDM2 can inhibit the transcriptional activity of p53, alter its subcellular localization and modulate its protein stability. The MDM2-p53 hub is also vital for dictating cell cycle arrest, and tumor suppression [135, 136]. TRIM-p53 interactions have been extensively investigated. In many studies of TRIMs, TRIMs were mainly shown to regulate p53 stability and activity. Conversely, the alteration of p53 results in transcriptional regulation of TRIM proteins such as TRIM22 [137, 138], TRIM3, TRIM8, TRIM19, TRIM24, TRIM32, and TRIM67 [139]. Because of the intricate roles of TRIMs in regulation of p53 stability, we simply divide TRIMs into several subgroups: positive regulators, negative regulators, and others that play unidentified role.

##### Negative regulators

The negative regulatory effect is mainly achieved by promoting ubiquitination of p53. A number of TRIMs has been confirmed thus far. TRIM23 was reported to physically interact with p53, thereby promoting its ubiquitination [51]. The TRIM24-p53 link was first discovered in embryonic stem cells [140]. TRIM24 acts as a negative regulator of p53 by ubiquitinating and promoting degradation. Moreover, a crucial negative feedback loop is formed between TRIM24 and p53. For example, TRIM24 could be destabilized by phosphorylation at S768 in response to DNA damage. In turn, the transcription of TRIM24 is directly induced by p53 [141]. Research has demonstrated that TRIM31 promotes anoikis resistance by targeting p53 for degradation. Additionally, TRIM32 also promoted p53 degradation through ubiquitination. Thus, TRIM32 could negatively regulate p53-mediated apoptosis, cell cycle arrest, and senescence in response to stress [142]. TRIM39 can directly bind and ubiquitinate p53 in vitro and in vivo, leading to p53 degradation [143]. TRIM47 also promoted RCC cell proliferation in vitro and in vivo by increasing p53 ubiquitination and degradation [144]. TRIM59 was also reported to be overexpressed in human gastric tumors, leading to decreased p53 levels and enhanced cell proliferation and metastasis [145]. Moreover, TRIM27 [146], TRIM69 [147, 148], and TRIM71 [149] were found to interact with p53 and induce its ubiquitination and degradation.

Some TRIMs function as vital negative regulators of p53 in various ways. HuR is an RNA-binding protein that activates p53 mRNA translation. TRIM21 has a role in fine-tuning p53 protein synthesis by modulating HuR protein levels in response to UVC-induced DNA damage in breast cancer [150]. Moreover, HuR binds to the 3’-UTR of TRIM21 mRNA and activates its translation, thereby constituting a negative feedback loop that may play a pivotal role in the p53 regulatory response to genotoxic stress [151]. GMPS (GMP synthetase), is required for USP7-mediated p53 stabilization [152]. In general, TRIM21 can destabilize p53 by mediating the ubiquitination and degradation of GMPS or HUR [150, 152]. In addition, research has indicated that TRIM21 inhibits cellular senescence in glioma by suppressing the p53-p21 pathway at the mRNA and protein levels [77]. p21 is a negative regulator of p53 stability. The p21 expression level was also negatively controlled by TRIM28 by promoting its Ubiquitination-mediated degradation [75]. Research has also shown that some TRIMs play oncogenic roles by downregulating p53: TRIM11 exerts its oncogenic effect in HCC by negatively regulating p53 expression [153]. TRIM29 was proven to directly bind p53 and alter p53 subcellular localization, inhibiting its transcriptional activation function, and promoting cancer cell proliferation [154]. The p53 protein expression level is also regulated by TRIM59 [155] and TRIM66 [156]. Further study is needed to support these conclusions and explore the specific mechanism.

##### Positive regulators

The positive regulation of p53 is achieved mainly by modulating the MDM2 (murine double minute 2) -p53 hub. TRIM8 participates in a regulatory pathway controlling p53. Under stress conditions, TRIM8 is recruited by p53, and plays a stabilizing role by physically directly interacting with p53 and inducing the degradation of the MDM2 protein, reducing the interaction between p53 and MDM2, and resulting in cell cycle arrest and a reduction in cell proliferation. In turn, p53 promotes the transcription of TRIM8 by binding to the responsive element in the first intron [157]. The overexpression of TRIM13 results in ubiquitination and proteasomal degradation of MDM2 and AKT, which increases p53 stability and decreased AKT kinase activity, eventually enhancing irradiation-induced apoptosis [158]. TRIM67 exerts a similar effect, while, binding with the C-terminal region of p53, disrupting the interaction between p53 and MDM2, and thereby stabilizing p53 protein levels to suppress colorectal cancer initiation and progression [127]. TRIM3 [159], TRIM52 [160], and TRIM58 [161] also affect p53/p21 in breast cancer. Apart from the ubiquitination pathway, TRIML2 can enhance p53 protein levels through SUMOylation in response to DNA damage [162]. TRIM19, also known as promyelocytic leukemia protein (PML), directly binds to p53 and induces its SUMOylation at lysine 386, leading to stabilization and enhanced transcriptional activity of p53 [163, 164]. TRIM29 could bind to Tip60, and reduce acetylation of p53 at lysine 120 by Tip60, promoting cancer cell proliferation and enhancing transformation [165]. TRIM14 was reported to activate C-myc, p53, and Bax gene expression during embryonic development to modulate apoptosis [166]. TRIM32 [167]. TRIM3, TRIM19, TRIM69, and TRIM71 were also demonstrated to be positive regulators [139, 168]. The specific mechanisms still need to be explored.

##### Others

Some members of the TRIM family may regulate p53 by diverse mechanisms, thus having both positive and negative roles. For instance, as mentioned above, TRIM31 directly interacts with p53, and induce its ubiquitination [169]. Moreover, TRIM31 suppresses the MDM2-mediated K48-linked ubiquitination of p53 by competitively inhibiting the interaction of MDM2 and p53, leading to p53 stabilization and activation of the p53-AMPK axis [80]. In addition to TRIM31, there are still some TRIM proteins that act as a double-edged sword or have an intricate role in p53 regulation including TRIM14, TRIM37, and TRIM40 [170].

Since a link between the TRIM family and p53 has been intensively studied, it is not surprising that TRIMs are critical for the regulation of p53 functions in cell fate specification and survival. There are many cases in which some TRIMs regulate the cancer cell cycle and cell growth through a p53-independent mechanism [149]. Other than p53, TRIMs are closely connected with some cell cycle regulatory proteins: TRIM6 was reported to induce cell cycle arrest by mediating the ubiquitination of TIS21 (12-O-tetradecanoyl phorbol-13-acetate inducible sequence 21), an antiproliferative factor involved in the regulation of G2/M arrest [171]. Another important cell cycle regulatory protein TDP43 (recombinant tar DNA binding protein 43 kDa) can be regulated by TRIM16 [74]. Depletion of TRIMs such as TRIM59 leads to the accumulation of cell in S-phase [172].

Major advances have been made in p53 drug discovery in recent years. Progress in both screening methods and structural biology has paved the way to address the many ways in which p53 is inactivated in cancer, although many challenges remain, especially in the case of p53 DNA-contact mutants. There are now more strategies for targeting aberrantly overexpressed p53 regulators in cancers with wild-type p53. Inhibiting the interaction of p53 with its negative regulators MDMX and MDM2 has proven to be the most successful thus far, with several inhibitors from both academia and industry currently in clinical trials [173]. Considering the intimate connection between TRIMs and p53, we believe that some TRIM family proteins could be optimal targets.

#### The JAK/STAT signaling pathway

The JAK/STAT pathway is known to be activated in IFN mediated antiviral activity. Owing to the important roles of JAK/STAT in cellular biology, subtle changes in their cellular concentrations could have important phenotypical consequences [174]. Constitutive activation of the JAK/STAT signaling pathway has been associated with malignancy since the 1990s. Mechanisms that cause JAK/STAT pathway activation in cancer could be activating mutations of receptors or STATs themselves and upstream oncogenes that in turn activate downstream factors or cytokine overexpression by autocrine/paracrine [175]. JAK/STAT signaling may contribute to cancer pathology by promoting inflammation, obesity, stem cells, and the premetastatic niche [176].

In physiological activities, TRIMs are closely related to the JAK/STAT signaling pathway. For example, in response to LPS, TRIM59 can recruit PIAS1 (protein inhibitor of activated STAT1) to bind with STAT1 to suppress its activation [177]. For cancer initiation and development, TRIMs may play a role through the JAK/STAT3 pathway: knockdown of TRIM66 reduced the activation of phosphorylation levels of JAK2 and STAT3 in CRC cells, and significantly inhibited cell proliferation, migration, and invasion of CRC cells [178]. TRIM8 interacts with SOCS-1 (suppressor of cytokine signaling 1), a negative regulator of STAT3 activation by the SH2 domain, and mediates its degradation, allowing the activation of JAK/STAT induced via Interferon [179]. TRIM14 also acts as a prognostic factor in PTC by promoting the ubiquitination of SOCS-1 [43]. Another study revealed that TRIM14 acts as a positive regulator of the SPHK1/STAT3 signaling pathway, promoting CRC cell migration and invasion [180]. Several TRIMs, such as TRIM27, TRIM29, and TRIM52, have also been reported to mediate the overactivation of STAT3 and induce the expression of downstream target genes, such as MMP-2 (Matrix metalloproteinases 2), MMP-9, and VEGF (vascular endothelial growth factor), thus promoting cancer cell migration and invasion [180–183]. In contrast, TRIM16 is involved in the JAK/STAT pathway as a negative factor in miR-135-regulated NSCLC sensitivity to gefitinib [184].

The antitumor immune surveillance processes orchestrated by the activity of immune cells and immune-regulatory factors are chiefly driven by JAK/STAT signaling [185]. Interestingly, TRIMs have also been widely reported to be associated with the innate immune response. TRIMs regulate the IFN-I mediated innate immune response, which plays a vital role in antiviral and antitumor responses [186]. The contributions of TRIMs to the dysregulation of the JAK/STAT pathway in cancer cells have been illustrated in several studies above. The connection between TRIMs, and JAK/STAT-associated antitumor immune surveillance has not been established. Therefore, the new research topic could be studied: TRIM-mediated antitumor immune response.

#### The PI3K/AKT signaling pathway

As a consequence of extensive downstream factors and complicated crosstalk with other signaling pathways, PI3K/AKT is involved in almost all cellular processes. Thus, anomalous activation is frequently detected in a range of tumor types. To date, more than 50 compounds that target key components of this signaling network have been tested in clinical trials involving patients with various cancers, indicating its importance in oncology [187].

The oncogenic functions of TRIM11 in cancer have been investigated in cholangiocarcinoma, HCC [188], lung cancer [92], ovarian cancer [189], and cervical cancer [190]. Downregulation of TRIM11 decreases the phosphorylation levels of p-PI3K and p-AKT. Furthermore, TRIM11 can facilitate the K48-linked ubiquitination-mediated degradation of PHLPP1 (PH domain and leucine rich repeat protein phosphatase 1), which suppresses AKT by dephosphorylation, activating the AKT signaling pathway [30, 130]. In addition, TRIM11 promoted glycolysis by modulating AKT/GLUT1 in breast cancer cells [191]. Overexpression of TRIM46 also increases glycolysis in LUAD cells and the cell proliferation rate, and promotes xenograft growth by increasing the ubiquitination of PHLPP2 and upregulating of p-AKT in lung cancer [97]. Downregulation of TRIM59 restricts the PI3K/AKT/mTOR signaling pathway [192]. TRIM44 also promotes the AKT pathway and affectes its downstream mTOR signaling pathway, modulating the EMT process [44, 193]. TRIM26 exerts an oncogenic effect in bladder cancer via AKT/GSK3β/β-catenin [194]. TRIM14 knockdown increases the protein levels of PTEN, which subsequently inactivates AKT pathways in melanoma [195] and colorectal cancer [196]. TRIM14 overexpression resultes in lower proliferation rates and reduced apoptosis in AML [197]. In addition, similar effects were observed in osteosarcoma [198] and gastric cancer [199]. TRIM37 was also identified as a modulator of the ubiquitination of PTEN, thereby activating the AKT/GSK-3β-β-catenin axis in pancreatic cancer [59, 200]. The TRIM27 expression level can be reduced by an AKT inhibitor [201]. TRIM27 functions as a pro-proliferative factor, and participates in energy metabolism by activating the PI3K/AKT pathway in esophageal squamous cell carcinoma [124], and cell cycle arrest, and apoptosis in ovarian cancer [202] and facilitates the invasion and metastasis of colorectal cancer [53]. TRIM59 was proven to enhance PC progression by activating the PI3K/AKT/mTOR-glycolysis signaling pathway [61], CRC [203], breast cancer [88], and cholangiocarcinoma progression [192]. Some TRIMs may exert their role by directly affecting AKT and downstream signaling pathways or being modulated by AKT. A previous study demonstrated the involvement of TRIM24 in the regulation of the AKT signaling pathway in bladder cancer [204]. A later study proved that TRIM24 could directly activate the PIK3CA gene, which encodes the catalytic subunit of PI3Ks in glioma [205]. To date, two TRIM family members, TRIM13 [158] and TRIM21 [206] have been identified to directly mediate the proteasomal degradation of AKT. TRIM21 can be negatively regulated by AKT [207]. In addition, TRIM31 [42, 208], TRIM32 [69], and TRIM47 [209] were reported to play a role in the AKT signaling pathway in cancer.

The clinical efficacy of PI3K inhibitors has been challenged by adverse clinical effects such as hyperglycemia and tumor resistance. Targeting AKT has great therapeutic potential but may also be limited owing to the extensive downstream substrates and complicated crosstalk with other signaling pathway networks [210]. Clarifying the relationship between TRIMs and PI3K/AKT may provide a new perspective on cancer therapy. Although the connection with multiple TRIM family proteins has been identified, studies have not fully elucidated the specific way that TRIMs affect the PI3K/AKT signaling pathway.

#### The TGF-β signaling pathway

The transforming growth factor β (TGF-β) signaling pathway controls the homeostasis of cell and tissue development. The transduction process of the TGF-β/SMAD pathway usually includes the initial binding of a TGF-β family member to its type II receptor in concert with a type I receptor. Then, the complex activates the type I receptor, and subsequently phosphorylates a receptor-regulated SMAD2/3, allowing it to associate with SMAD4 and to move into the nucleus to bind to specific enhancers in target genes activating transcription [211]. The altered production of, or responsiveness to, TGF-β changes cancer cell behavior. Interestingly, the TGF-β signaling pathway is now considered a double-edged sword in which TGF-β exerts both positive and negative influences on cancer development.

The connection between TRIMs and TGF-β has been identified in several articles. Endoglin is an auxiliary component of the TGF-β receptor and has been shown to have a binding relationship with TRIM21 in human endothelial cells, although further regulatory mechanisms have not been revealed [212]. TRIM33 functions in the TGF-β signaling pathway by interacting with SMAD2/3 and ubiquitinating SMAD4 [213, 214]. TRIM47 also physically interacts with SMAD4, increasing its ubiquitination and degradation [215]. TRIM37 promotes RCC cell EMT and malignant progression through TGF-β signaling activation, as a consequence of TRIM37 mediated H2A ubiquitination modification [39]. TRIM59 was found to downregulate the protein expression of p-SMAD2 and thus inhibit the activity of the TGF-β signaling pathway [216–218]. Likewise, in bladder cancer, silencing TRIM59 inhibites the TGF-β/SMAD2/3 signaling pathway and affectes the downstream EMT process [217]. TRIM62 promotes the ubiquitination of SMAD3, acting as a negative regulator in the TGF-β pathway. Thus, it also influences the downstream targets of AMAD2-SNAL1/2 and the EMT process [219]. The downregulation of TRIM52 significantly induces SMAD2/3 dephosphorylation in hepatocellular carcinoma cells [160]. Research also revealed that TRIM66 [220], TRIM25 [221–223] and TRIM32 are involved in TGF-β signaling pathways to promote cancer cell proliferation and invasion [68].

TRIM family proteins may positively or negatively regulate the TGF-β signaling pathway in cancer cells. However, due to the complexity of the TGF-β signaling pathway in regulating cancer cell activity. TRIMs may act as either tumor suppressors or promoters depending on the regulatory mechanisms and different cancer cell types. Therefore, further analyses are required to determine its roles in cancer initiation and progression.

#### The Wnt/β-catenin signaling pathway

The Wnt signal transduction cascade is a key driver of various tissue stem cells and the main regulator of development phases. Hence, mutated or dysregulated Wnt pathway components cause many growth-related pathologies and tumorigenesis, tumor progression and resistance [224]. To date, only a few TRIM family proteins have been associated with the abnormalities in the Wnt/β-catenin signaling pathway.

Glycogen synthase kinase 3β (GSK-3β) is a crucial upstream suppressor of β-catenin. Research has found that GSK-3β mediates the phosphorylation of β-catenin, promoting its ubiquitination [225]. Disheveled (Dvl), is a cytoplasmic protein that is activated when Wnt ligands bind to the coreceptor. Subsequently, activated Dvl attaches to the Axin/GSK-3β complex and antagonizes GSK-3β-dependent phosphorylation of β-catenin. TRIM29 can interact with Dvl-2 to stabilize β-catenin and exert oncogenic functions in pancreatic cancer [226, 227]. In addition, knockdown of TRIM29 enhances GSK-3β protein expression and inhibited the expression of β-catenin and c-myc, thereby activating the β-catenin signaling pathway in cervical cancer [228]. In colorectal cancer, TRIM29 induces the activation of the Wnt/β-catenin signaling pathway via upregulation of CD44 expression [126, 229, 230]. TRIM26 exerts an oncogenic role in bladder cancer through the regulation of cell proliferation, migration, and invasion via the AKT/GSK3β/β-catenin pathway [231]. TRIM32 could significantly enhance the expression of β-catenin and downstream target mRNAs in GC [232]. The latter study showed that TRIM32 ubiquitinates Axin1 (axis inhibition protein 1), which is a robust negative regulator of regulating Wnt/β-catenin signaling activity in rat nucleus pulposus cells [233]. TRIM46 was also found to act as a promoter in the ubiquitination and proteasomal degradation of Axin1 protein in HK-2 cells [234]. Silencing TRIM44 in PTC inhibited the proliferation, migration, and invasion of cancer cells by suppressing of the Wnt/β-catenin signaling pathway, indicating its oncogenic function [193]. TRIM31 promotes AML cell proliferation and sensitivity to daunorubicin in AML cells through the Wnt/β-catenin signaling pathway [235]. TRIM59 also has a positive role in the Wnt/β-catenin signaling pathway in neuroblastoma [236]. In contrast, TRIM58 showed a tumor-suppressive role by inactivating of β-catenin signaling via ubiquitination in gastric cancer [237]. Regarding tumor resistance, TRIM11-mediated drug resistance depends on activating β-catenin/ABCC9 signaling by facilitating the degradation of Daple in NPC [46]. Moreover, TRIM15 [238], TRIM24 [32], TRIM37 [239], and TRIM47 [240] were demonstrated to positively modulate β-catenin pathways.

In summary, based on all these studies, the majority of TRIM family proteins appear to act as oncogenic factors when they activate the Wnt/β-catenin signaling pathway. However, whether the aberrant Wnt/β-catenin signaling pathway regulated by TRIMs contributes to cancer stem cell formation and development is still unknown. Nevertheless, the additional regulatory mechanisms that have been identified for the control of β-catenin help us better understand why the aberrant Wnt/β-catenin signaling pathway exerts crucial roles in cancer stem cell renewal, cell proliferation, and differentiation.

#### The NF-κB signaling pathway

The importance of NF-κB signaling in the cancer field has been understood for decades. It has become increasingly clear that the anomalies in the NF-κB signaling pathway are involved in cancer development and progression, as well as in resistance to chemotherapy and radiotherapy [241, 242]. Moreover, many reports have suggested that TRIM family proteins are recruited at different steps of TNF-α-induced activation of the NF-κB signaling pathway and form a feedback loop in the innate and adaptive immune pathways [243].

In osteosarcoma, TRIM10 contributes to cisplatin resistance by increasing the nuclear levels of p65, thereby activating canonical NF-κB signaling [244]. TRIM8 could activate the NF-κB signaling pathway in two ways: promoting the cytoplasmic degradation of PIAS3 (protein inhibitor of activated STAT3), inhibiting its binding with p65 [245]; and mediating the Lys63-linked polyubiquitination of TAK1 (a serine/threonine kinase essential for TNFα- and IL-β-induced NF-κB activation) at Lys158, activating downstream NF-κB [246]. Interestingly, the atypical TRIM family protein TRIM44 which lacks the RING finger domain, was revealed to promote breast cancer cell proliferation and migration by enhancing NF-κB signaling [84]. Further study revealed that TRIM44 knockdown substantially attenuated the TNFα-dependent phosphorylation of the p65 subunit of NF-κB and IκBα [85]. IκBα, as an inhibitor of NF-κB, can interact with TRIM27 [34] and TRIM22 [247] to achieve ubiquitination-mediated degradation, leading to NF-κB activation. Further research found that TRIM22 accelerated IκBα degradation by inducing K48-linked ubiquitination. TRIM22 also forms a complex with the NF-κB upstream regulator IKKγ and promotes K63-linked ubiquitination, which leads to the phosphorylation of both IKKα/β and IκBα [248]. A negative regulator of the NF-κB signaling pathway-, TRIM67, was demonstrated to competitively bind β-TrCP (β-transducin repeat-containing protein) to IκBα, resulting in inhibition of β-TrCP-mediated degradation of IκBα, which finally resulted in inhibition of TNFα-triggered NF-κB activation [249]. In LUAD, elevated TRIM23 expression is positively correlated with expression of the NF-κB [95]. A link between TRIM37 and the NF-κB pathway was described in the context of NSCLC where NF-κB is constitutively activated. Related research has demonstrated that TRIM37 induces the K63 polyubiquitination of TRAF2 (TNF receptor associated factor 2), a significant activator of NF-κB signaling [250]. PKC-ε (protein kinase C-ε), which also acts as an NF-κB-activating protein kinase, is directly associated with TRIM47 to achieve lysine 27-linked polyubiquitination of PKC-ε, enhancing NF-κB signaling, which in turn up-regulates TRIM47 [86]. In addition, TRIM46 acts as an oncogene in OS by interacting with and ubiquitinating PPARα (peroxisome proliferator-activated receptor α), leading to the activation of the NF-κB signaling pathway [251]. Activation of the NF-κB pathway is also positively correlated with TRIM52 [252], TRIM14 [253], and TRIM31 [254] and promotes cancer development in cancers. In NSCLC, TRIM13 behaves as a tumor suppressor through by negatively regulating the NF-κB pathway [255].

The relationship between TRIM family proteins and NF-κB signaling transduction has been investigated mainly in immune response research. Further studies are needed to help clarify the role of TRIM-mediated regulation of NF-κB in different pathological conditions, especially in antitumor immunity and tumor resistance.

#### The notch, AMPK, MAPK, and YAP signaling pathway

The Notch, AMPK, MAPK, and YAP signaling pathways have also been reported to play vital roles in cancer. Knockdown a crucial coactivator of Notch receptors and signal transmission and function, decreases the expression of TRIM13, TRIM32, TRIM44, and TRIM59 especially in T-ALL [94]. The activation of MAPK enhances TRIM2 protein levels and affectes its binding with Bim, resulting in reduced Bim in tamoxifen-resistant breast cancer cells [256]. TRIM28 also regulates the degradation of AMPK [257]. TRIM22 inhibites osteosarcoma progression by promoting proteasomal degradation of NRF2 independent of KEAP1, thereby activating AMPK/mTOR/autophagy signaling that led to autophagic osteosarcoma cell death [101]. In colorectal cancer, TRIM24 directly interactes with the YAP promoter at the 983 to 734 site and activated YAP transcription, ultimately enhancing the proliferation of cells [258]. In breast cancer, TRIM6 was demonstrated to interact with STUB1 (STIP1 homology and U-box containing protein 1) and mediate its degradation thereby promoting YAP1 signaling [259]. TRIM31 promotes the malignant behaviors of HCC cells by directly promoting the E3 ligase-mediated K48-linked ubiquitination and degradation of upstream suppressors of the mTORC1 pathway [35].

In summary, the TRIM family and the involved signaling pathway do not have a simple one-way relationship. In fact, the same TRIM molecule may directly or indirectly participate in multiple signaling pathways, acting both as an oncogene and a tumor suppressor gene. Additionally, one signaling pathway may require multiple molecules to precisely regulate each step of the cell life cycle. Understanding the regulatory network of the TRIM family is the basis for developing related therapies.

### TRIM family and tumor resistance

Owing to the heterogeneity of various types of malignancies and the complicated tumor microenvironment, tumor resistance has been a difficult topic for decades. Based on the studies thus far, we summarized and subdivided the drug resistance based on different mechanisms. The mechanisms of TRIMs in cancer development and therapy resistance can mainly be classified into epithelial-mesenchymal transition (EMT), cancer stem cells (CSC) theory and other theories. The resistance mechanisms are also presented in Fig. 3.

#### EMT

EMT is a reversible cellular programmer that transiently places epithelial cells into mesenchymal cell states. During this process, epithelial cells progressively lose their cobblestone epithelial appearance in monolayer cultures to adopt a spindle-shaped, mesenchymal morphology [260, 261]. EMT was found to be activated during cancer progression and attributed to cancer resistance. TRIM directly or indirectly modulates EMT through various signaling pathways. For example, TRIM50 acts as a tumor suppressor in HCC cells by directly targeting snail and reversing EMT [28]. TRIM50 also inhibits pancreatic cancer progression and reverses EMT [56]. TRIM24 [32] and TRIM58 [50] are involved in EMT by modulating snail, slug, and vimentin, and E-cadherin. The effect of TRIM11 in promoting the EMT process has been confirmed in hepatocellular cancer [188], gastric cancer [262], and lung cancer [92]. TRIM65 modulates cytoskeleton rearrangement and induces UCB cell EMT by the ubiquitination of Annexin A2, ultimately leading to enhanced invasiveness of UCB cells [41]. ZEB2 (zinc finger E-box binding homeobox 2), a transcription factor involved in EMT, is also a downstream target of TRIM14. The overexpression of TRIM14 promotes EMT through ZEB2 [76]. Interestingly, knockdown of TRIM16 accelerates EMT by inhibiting ZEB2 expression, which in turn inhibits the transcription of E-cadherin [31]. TRIM16 was also proven to suppress the sonic hedgehog signaling pathway as well as the EMT process in ovarian cancer [263]. TRIM62 functions as a significant regulator of apical-basal polarity and acinar morphogenesis, as well as a chromosome 1p35 tumor suppressor and negative regulator of TGFβ-driven EMT [219]. TRIM29 also induced an EMT phenotype in PDA by the Wnt/β-catenin pathway [126]. Apart from the main pathway mentioned above, TRIM21 [128], TRIM27 [53], and TRIM66 [178], were also related to the EMT process. Nevertheless, in-depth research on the detailed mechanisms is needed, and the studies above provide new insights into the progression of cancer.

#### CSCs

The CSCs theory is a classic cancer resistance mechanism, which suggests that CSCs are inherently resistant to some cancer therapies [264]. Within a heterogeneous tumor mass, only approximately 0.05–3% of cells are suspected to be CSCs. Interestingly, CSCs, cancer cells, and normal stem cells share a variety of cytochemical properties. For example, inhibition of the redox system for ROS production and a high drug efflux property, which contribute to resistance to anticancer drugs. CSCs can also undergo EMT transition to better migrate to the surrounding tissues [265]. With the concept of CSCs evolving, researchers have found that the abnormalities in TRIM family activities function in the acquisition and maintenance of the cancer stem cell phenotype. TRIM29 modulates the CSC-like features of PDAC [266]. A recent study identified TRIM29 maintenance of CSCs-like characteristics in ovarian cancer through facilitated SETBP1 (SET Binding Protein 1) transcriptional activation and the downstream SET axis [267]. TRIM37 was also able to promote PC stem cell-like traits [59]. TRIM6, TRIM11, TRIM14, TRIM19, TRIM24, TRIM25, TRIM27, TRIM28, and TRIM71 act as positive regulators in the acquisition and maintenance of CSCs. TRIM16, TRIM21, and TRIM32 act as negative regulators [268]. Another study found that TRIM16, TRIM32, TRIM24, TRIM8, TRIM27, TRIM11, and PML are associated with cancer stem cells and the clinical prognosis of RCC, and affect tumor progression [269]. The DNA damage and repair (DDR) process determines the fate of cell death and survival under endogenous and exogenous sources of pressure. In some cases, DNA damage is not addressed properly, leading to genome instability, which makes the cell susceptible to malignant transformation. Many proteins are involved in this signaling cascade including TRIMs. A subfamily of the TRIM-transcriptional intermediary factor 1 (TIF1) family consists of TRIM24, TRIM48, TRIM33, and TRIM66 [12]. The TIF1 family plays an important role in DNA repair and mitosis, transcription, cell differentiation and cell cycle regulation, and immunity. The TIF1 family members are often dysregulated in hepatocellular carcinoma [270], manifested as altered gene expression, deletions, translocations, or loss-of-function mutations [12]. Interestingly, the TIF1 family exhibits distinct expression patterns in stem cell-like tumors. In contrast to the rest of the TIF1 members, only TRIM28-associated gene expression profiles are strongly enriched with stemness markers regardless of the tumor type. Moreover, TRIM28 is highly expressed in higher-grade tumors with stem cell-like traits [271]. Overall, we still need more evidence to explore and support TIF1 function in maintaining genome stability during DNA damage in cancer stem cells. To date, CSCs developing chemoresistance during chemotherapy regimens remain the hardest obstacle to overcome for cancer treatment, and TRIMs may be potential targets.

#### Other mechanisms

TRIM family is involved in chemoresistance in many cancers. Since substantial TRIM family proteins mediate the chemotherapy response and most of them function as a promoter to chemoresistance, there may be a new promising therapeutic target in the future. Table 2 summarize the roles of TRIM family members in mediating cancer cells to chemotherapy and mechanisms.

**Table2:** The roles and mechanisms of TRIM family proteins in mediating chemoresistance

TRIM family	Cancer types	Chemotherapy to	Mechanisms	References
Overexpression of TRIM7	Osteosarcoma	DOX (Doxorubicin hydrochloride) MTX (Methotrexate)	Promotes the Ubiquitination of BRMS1 (breast cancer metastasis suppressor 1)	[102]
Downregulation of TRIM10	Osteosarcoma	Cisplatin	activates the NF-κB signaling pathway	[244]
Overexpression of TRIM58	Breast cancer	DOX	inactivates p53/p21 axis	[161]
Overexpression of TRIM8	CRC and ccRCC	Cisplatin	Inhibits the stability and activity of P53	[272]
Overexpression of TRIM24	Gastric cancer	5-FU	Upregulates cyclin D1 and Akt phosphorylation	[273]
Overexpression of TRIM17 or Downregulation of TRIM28	Melanoma	DOX	Reduces BCL2A1 (B-cell lymphoma 2-related protein A1) protein levels	[274]
Downregulation of TRIM72	Uveal melanoma	Dacarbazine (DTIC)	Inhibits ubiquitination of MGMT (O6-methylguanine DNA methyl transferase)	[275]
Overexpression of TRIM29	LUAD	Cisplatin	NA	[276]
Overexpression of TRIM23	LUAD	Cisplatin	Regulates glucose metabolism via NF-κB/GLUT1/3 axis	[95]
Overexpression of TRIM46	LUAD	Cisplatin	Enhances glycolysis	[97]
Downregulation of TRIM65	NSCLC	Cisplatin	Regulates miR-138-5p	[277]
Overexpression of TRIM59	NSCLC	Cisplatin	Regulates PTEN/AKT	[278]
Downregulation of TRIM72	NSCLC	Cisplatin	NA	[279]
Overexpression of TRIM11	Nasopharyngeal carcinoma	Multidrug	Inhibits apoptosis and modulates the Daple/β-catenin	[46]
Overexpression of TRIM37	Pancreatic Cancer	5-FU	Promoted promotes ubiquitination of PTEN and activates of the AKT-GSK-3β-β-Catenin	[59]
Overexpression of TRIM31	AML	Daunorubicin	Regulates Wnt/β-catenin pathway	[235]
Downregulation of TRIM21	Glioma	Temozolomide	NA	[77]
Overexpression of TRIM31	Glioma	Temozolomide	Activates PI3K/Akt	[280]
Overexpression of TRIM47	CRC	5-FU	Promotes ubiquitination of SMAD4	[215]
Overexpression of TRIM8	CRC	Chemotherapy	NA	[245]

The varied expression level of different TRIM family proteins are positively related to the chemotherapy resistance in different types of cancer. The mechanisms are listed in the table as well fluorouracil.

*NA* not applicable

In addition to chemotherapy, targeted cancer therapy mainly consists of two approaches: antibodies and small molecules. Most of them are highly selective with fewer side-effects than chemotherapy. Although many drugs have achieved success in clinical trials, drug resistance remains a challenge. Several studies have identified the role of TRIMs in targeted therapy: The sensitivity of NCSLC cells to gefitinib is mediated by TRIM16, as the suppression of miR-135 could improve sensitivity to gefitinib by upregulation of TRIM16 [184]. A study found that apatinib-reduced gastric cancer cell proliferation was significantly abolished by TRIM21 knockdown; in turn, promoting TRIM21 expression further improved the sensitivity of gastric cancer cells to apatinib [63]. In addition, the sorafenib resistance in HCC cells could be mediated by TRIM37 overexpression [36]. EBBP (estrogen-responsive b box brotein) is also a member of the TRIM protein family. EBBP acts to derepress the transcription of RARb2 (retinoic acid receptor β2) and CYP26A1 (cytochrome P450 family 26 subfamily A member 1), by modifying histone acetylation in retinoid-resistant cancer cells [281].

In hormone-driven cancers such as prostate cancer, ligand-deprivation therapy is used as a standard treatment. Sometimes it fails when tumors evolve to activate androgen receptor signaling. Several TRIMs are involved in steroid hormone regulation [282]. TRIM24 was deemed a major effector of “AR reprogramming” [283] and contributed to castration-resistant prostate cancer (CRPC) [283]. In ER^+^ breast cancer, TRIM39 [83] and TRIM29 knockdown significantly suppresse ER^+^ breast cancer cell proliferation [79]. In addition, in ER^+^ breast cancer, TRIM3 promotes SUMO modification of estrogen receptor 1(ESR1) and activated the ER signaling pathway. TRIM3 also confers tamoxifen resistance [82]. TRIM2 is also involved in tamoxifen resistance through binding with Bim [256]. TRIM47 facilitates endocrine therapy resistance in breast cancer by affecting the NF-κB signaling pathway [86]. In addition to these findings, the overexpression of TRIM29 was shown to mediate the resistance to ionizing radiation (IR) [154]. A recent study revealed that the TRIM29 and TRIM37 genes were involved in the cell response to radiation and could function as predictive biomarkers for radiation sensitivity in normal and tumor cell lines [284].

The therapeutic resistance of cancer is an evolving paradigm as novel regimens merge from chemotherapy, and target therapy to immune therapy. By virtue of advanced screening techniques and biological approaches, novel mechanisms of drug resistance and molecular signatures and genotypes have been gradually uncovered to help us better predict tumor responses and resolve resistance.

## The therapeutic potential of TRIM family

Since the late 1990s, clinical trials of small molecular inhibitors have been conducted for a variety of malignancies. Although these drugs have dramatically improved outcomes, the phenomenon of tumor resistance and relapses remain and difficult to overcome [285]. Moreover, the absence of catalytic sites limits the development of covalent inhibitors and high-affinity inhibitors.

For the past few years, the antitumor activity of protein-targeting chimeric molecules (PROTACs), which induce proteasome-mediated degradation of specific protein targets is being evaluated clinically. Overall, E3 ubiquitin ligase-based drug discovery research is moving toward a promising future in its third decade, as there are now many PROTACs and E3 ubiquitin ligase-based small molecule drugs in the pipeline from multiple biotechnology startups or companies at different preclinical and clinical stages. It will be a crucial decade for UPS and E3 ligase-based drug discovery. However, there are no reports of invention of TRIM-related PROTACs. The main bottleneck is the lack of structural information and the multiplicity of functions and mechanisms of the TRIM family in cellular activities [286]. Recently, a “Trim-Away” technique discovered in 2017, which rapidly and selectively degrade endogenous proteins without prior modification also opens a new window. The researchers harnessed TRIM21 which possesses high-affinity antibody-binding activity to drive the degradation of endogenous proteins by introducing exogenous TRIM21 and an antibody against the protein of interest [287]. Another team have also successfully exploited off-the-shelf antibodies and TRIM21 to carry out rapid protein depletion [288]. A Nano-ERASER based on this concept also successfully degrades COPZ1 (coatomer protein complex ζ1COPZ1), a vital protein in cancer cells [289]. Other than TRIM21, Some TRIM family members such as TRIM5 who shares many of the same ubiquitination characteristics with TRIM21 may also be eligible targets in the TRIM-Away field. This technique is promising in providing a tool for endogenous protein function study as well as paves the way for novel therapeutic drugs for cancer.

We have talked the mechanisms of TRIM family in cancer development and mediating therapy resistance. However, there are not much progress in directly regulating the level of TRIM members in cancer. Currently, there is only a chimeric antibody against TRIM14 was shown to inhibit osteosarcoma aggressiveness through the NF-κB signaling pathway [253]. So far, several critical questions need to be addressed at present: the structure and function of some TRIM family members are not clearly understood; The downstream direct substrates and upstream regulators of TRIM family members are largely elusive. The clinical significance of TRIMs should be highlighted by more mechanism research and development of detection techniques; Discovering potential biomarkers and drug targets through plasma proteomics and fundamental clinical study. Suppressing the oncogenic protein by small molecules or pro-degradation have achieved great success while blocking one signaling pathway is not sufficient to obtain the desired therapeutic outcome. Tumor cells possess an enhanced ability to degrade misfolded proteins through upregulation of the TRIM proteins [113]. Consequently, targeting TRIM family to inhibit their activity may also be a new strategy for cancer therapy.

## Conclusions

This review highlights the important roles of TRIMs in cancer initiation, development, and progression, and conducts an intensive and comprehensive investigation of the potential clinical implications and regulatory mechanisms by which TRIMs contribute to the dysregulation of various signaling pathways including JAK/STAT, PI3K/AKT, TGF-β, NF-κB, Wnt/β-catenin, and p53. TRIMs are also involved in tumor resistance and modulating the EMT process and acquisition and maintenance of CSC phenotypes. Moreover, the TRIM family could play important roles as cancer biomarkers and prognostic factors [290]. Hence, the combined effect and potential value of TRIM proteins in the clinic need further extensive research. In the end, we also talked about the therapeutical potential of TRIM family which we believe is promising especially in the “TRIM-away” filed.

To conclude, despite recent advances in illuminating the involvement of the TRIM family in cancer pathology, and developing innovative reagents and methodologies for cancer treatment, there are still many conundrums that need to be addressed. Further work is needed to identify and explore the potential of TRIM proteins to serve as therapeutic targets.

## Data Availability

Not applicable.

## Abbreviations

***DEAR1***: Ductal epithelium-associated RING chromosome 1
***IFN-I***: Type I Interferons
***Iκbα***: Iinhibitor of NF-κB
***PI3K***: Phosphoinositide 3-kinase
***Keap1***: Kelch like ECH associated protein 1
***DEN***: N-nitrosodiethylamine
***3D***: Three-dimensional
***PKD3***: Protein kinase C epsilon (PKC-ε) and protein kinase D
***ELFN1-AS1***: Repeat and fibronectin type III domain-containing 1-antisense RNA 1
***TAK1***: TGFβ activated kinase 1
***T-ALL***: T cell acute lymphoblastic leukemia
***PPARα***: Proliferator-activated receptor alpha
***MAGEA3/6***: Melanoma antigens A3/6
***β-TrCP***: β-transducin repeat-containing protein
***PKC-ε***: Protein kinase C epsilon
***PKD3***: Protein kinase D3
***PDX***: Patient-derived xenografts
***BCL2A1***: BCL2 related protein A1
***MCL-1***: Myeloid cell leukemia-1
***GLUT1***: Glucose transporter 1
***RPRD1A***: Nuclear pre-mRNA domain-containing protein 1A
***ESCC***: Esophageal squamous cell carcinoma
***ATDC***: Ataxia telangiectasia group D-complementing
***EGFR***: Epidermal growth factor receptor
***STAT3***: Signal transducer and activator of transcription 3
***NF-κB***: Nuclear factor κB
***RFP2***: Ret finger protein 2
***MAML1***: Mastermind-like protein 1
***T-ALL***: T-cell acute lymphoblastic leukemia
***HBE***: Normal human bronchial epithelial
***RCC***: Renal cell carcinoma
***PDCD10***: Programmed cell death protein 10
***RAR***: Dysregulating retinoic acid receptor
***AML***: Acute myeloid leukemia
***TGCT***: Testicular germ cell tumor
***LUAD***: Lung adenocarcinoma
***PHLPP2***: Pleckstrin homology domain leucine-rich repeat protein phosphatase 2
***NPC***: Nasopharyngeal carcinoma
***Daple***: Dvl-associating protein
***PDACs***: Pancreatic ductal adenocarcinomas
***SPHK1***: Sphingosine kinase 1
***VEGF***: Vascular endothelial derived growth factor
***STUB1***: Stress induced phosphoprotein 1 homology and U-box containing protein 1
***DDX3***: Dead-box RNA helicase 3
***GMPS***: Guanine monophosphate synthase
***SOCS1***: Cytokine-signaling-1
***TSC***: Tuberous sclerosis complex
